# Renewed coexistence as a conceptual reframing of animal reintroductions to foster sustainable human–wildlife coexistence

**DOI:** 10.1111/cobi.70195

**Published:** 2026-01-06

**Authors:** Roger Edward Auster, Alan Puttock, Stewart Barr, Richard Brazier

**Affiliations:** ^1^ Centre for Resilience in Environment, Water and Waste University of Exeter Exeter UK; ^2^ Geography Department University of Exeter Exeter UK

**Keywords:** coexistence, human–wildlife conflict, human–wildlife interactions, reintroduction, renewed coexistence, socioecological systems, translocation, coexistencia, coexistencia renovada, conflicto humano‐fauna, interacciones humano‐fauna, reintroducción, reubicación, sistemas socioecológicos, 共存, 人兽冲突, 人类与野生动物互作, 重引入, 迁地, 重新共存, 社会生态系统

## Abstract

Wildlife reintroductions are socioecological processes entailing the intentional movement of organisms by people. In animal reintroductions, there is growing recognition of the importance of human dimensions and efforts to integrate these into reintroduction projects. To conceptually reframe reintroductions as processes of renewed coexistence (a coadaptive process through which sustainable human–wildlife interactions [HWIs] are fostered), we build upon existing understanding of HWIs and coexistence. Our conceptual framing acknowledges historical HWIs and recognizes that the reintroduced species may be new for people to coexist with today. This provides a long‐term, futures‐oriented perspective on reintroductions that goes beyond the return of an animal to fostering long‐term coexistence between humans and the reintroduced animal. This requires integration of social understandings and meaningful involvement of people from the outset and throughout feasibility, planning, and implementation. Further, we provide fresh insight on the subsequent transition phase by recognizing there to be a period where humans and reintroduced animals continue to coadapt as the situation transitions from a reintroduction project into a long‐term coexistence between humans and wild animals.

## Abstract (es)

La renovación de la coexistencia como un marco conceptual de la reintroducción animal para promover la coexistencia humano‐fauna sustentable

**Resumen:**

Las reintroducciones de fauna silvestre son procesos socioecológicos que implican el traslado intencional de organismos hecho por personas. En las reintroducciones de animales, cada vez se reconoce más la importancia de las dimensiones humanas y los esfuerzos por integrarlas en los proyectos de reintroducción. Para replantear conceptualmente las reintroducciones como procesos de coexistencia renovada (un proceso de coadaptación a través del cual se fomentan las interacciones humano‐fauna sostenibles), nos basamos en los conocimientos existentes sobre las interacciones y la coexistencia entre los seres humanos y la fauna. Nuestro marco conceptual reconoce las interacciones históricas entre los seres humanos y la fauna y admite que las especies reintroducidas pueden ser nuevas para las personas con las que conviven hoy en día. Esto proporciona una perspectiva a largo plazo y orientada al futuro sobre las reintroducciones que va más allá del retorno de un animal para fomentar la coexistencia a largo plazo entre los seres humanos y el animal reintroducido. Esto requiere la integración de los conocimientos sociales y la participación significativa de las personas desde el principio y a lo largo de la viabilidad, la planificación y la implementación. Además, ofrecemos una nueva perspectiva sobre la fase de transición posterior, al reconocer que hay un período en el que los seres humanos y los animales reintroducidos siguen una coadaptación a medida que la situación pasa de ser un proyecto de reintroducción a una coexistencia a largo plazo entre los seres humanos y los animales silvestres.

## 将重新共存作为动物重引入的概念重构, 以促进可持续的人类与野生动物共存

**【摘要】** 野生动物重引入是一个社会生态过程, 需要人们有意识地移动生物。在动物重引入过程中, 人们越来越认识到人类因素的重要性, 并努力将这些因素纳入重引入项目。我们在对人类与野生动物互作和共存的现有理解的基础上, 将重引入项目在概念上重新定义为“重新共存的过程”, 即一个促进人类与野生动物可持续互作的共同适应过程。我们的概念框架承认人类与野生动物之间历史上的互作, 并认识到重引入物种对于当下的人类来说可能是新的共存物种。这为重引入提供了一个长期的、面向未来的视角, 超越了动物回归的范畴, 而是旨在促进人类与重引入动物之间的长期共存。这就要求从项目一开始和在整个可行性、规划及实施过程中, 都纳入社会理解和人们有意义的参与。此外, 我们还对随后的过渡阶段提出了新的见解, 认为在从重引入项目转变为人类与野生动物长期共存的过程中, 会存在一段人类与重引入动物持续共同适应的时期。**【翻译:胡怡思;审校:聂永刚】**

## INTRODUCTION

Wildlife reintroductions are a social process, as well as an ecological process. They are deliberate translocations of organisms to an area where the species was historically present but was extirpated (Berger‐Tal et al., 2020; IUCN SSC, 2013; Seddon et al., 2007). As human‐initiated interventions, reintroductions require a conscious choice to release individuals into present day socioecological contexts and are popular initiatives that often garner broad public support (Gaywood, 2024; IUCN SSC, 2013; Sampson et al., 2020). Whether for species conservation, to restore ecological function, to provide nature‐based solutions to social or environmental challenges, or for ideological or political reasons (Marino, McDonald, et al., 2024; Puttock et al., 2025; White et al., 2023), the restoration of species requires its long‐term survival in a landscape to be shared with humans.

In animal reintroductions, ecological factors, such as habitat availability and quality, have been a focus and success is often quantified based on ecological metrics (Marino, McDonald, et al., 2024; Seddon, 2015). Although these factors are central to reintroduction outcomes (Armstrong & Seddon, 2008), human dimensions have received less attention. Where efforts exist to include human factors, they have largely been restricted to capturing opinions without meaningful consideration of their deeper implications or relevance to policy and practice (Dando et al., 2023; Glikman et al., 2022).

Yet, social dimensions have considerable bearing upon the likelihood of success (Auster, Puttock, et al., 2020; Glikman et al., 2022; O'Rourke, 2014). The animal is not just being released into an ecological setting; it is being released into an anthropogenic landscape associated with human values, beliefs, behaviors, and management practices that are influenced by political, ethical, philosophical, and economic determinants (Glikman et al., 2022, 2023). Processes through which reintroductions are developed, hence, intersect across dimensions in a socioecological system—where the social and ecological are nested and interact (Binder et al., 2013; Folke et al., 2016; Orrick et al., 2024).

Failure to reflect upon social aspects can affect not only the likelihood of achieving objectives but also social dynamics, relationships between groups (Bavin et al., 2023; O'Rourke, 2014), and future environmental endeavors (Auster, Frith, et al., 2023a; Coz & Young, 2020; Gaywood, 2024). This is particularly relevant to reintroductions in which engagement with interest holders is perceived to have been insufficient (Coz & Young, 2020; Lute & Gore, 2014; Whitehead, 2025). Early integration of human dimensions will be more likely to prevent conflicts escalating and becoming increasingly difficult to resolve (Cusack et al., 2021; Glikman et al., 2022; Zimmermann et al., 2020), thus enabling better outcomes (O'Rourke, 2014; Serota et al., 2023).

Recognition of human dimensions is growing, and an increasing number of reintroduction projects have a social component (Brewitt, 2025; Dando et al., 2023; Serota et al., 2023). The International Union for Conservation of Nature (IUCN) advocates for social considerations in its *Guidelines for Reintroductions and Other Conservation Translocations* (IUCN SSC, 2013), often seen as the standard for conservation reintroductions. Others have sought to expand upon these guidelines to support deeper social understanding (e.g., Consorte‐McCrea et al., 2022; Glikman et al., 2023).

These are positive developments that enable learning that can be applied across contexts. Nevertheless, with rapidly growing interest and an increasing number of reintroductions globally (Berger‐Tal et al., 2020; Bubac et al., 2019; Taylor et al., 2017), we believe there is urgent need for a fundamental conceptual reframing of reintroductions to ensure human dimensions are anchored at their heart (Devine‐Wright et al., 2022), alongside ecological dimensions, to enable long‐term, socioecological success.

We advocate for a reframing through a human–wildlife interaction (HWI) lens and for application of such a framework before, during, and after the reintroduction. In so doing, we not only encourage an integrated research agenda across disciplines, particularly in the social sciences (including political science, geography, anthropology, psychology, and sociology [Devine‐Wright et al., 2022]) and ecological sciences, but also urge practitioners to develop approaches to reintroduction that foster sustainable shared landscapes in the long term.

## HWIs, COEXISTENCE, AND RENEWED COEXISTENCE

Predominantly, HWI research focuses on human–wildlife conflicts (HWCs) (Bhatia et al., 2020). HWC refers to conflicts between humans and wildlife—whether they are observable or are perceived to occur (Madden, 2004; Nyhus, 2016; Redpath et al., 2015). Many HWCs arise between people about wildlife, particularly where there are competing values or perspectives on environmental management and governance (Redpath et al., 2015). Hence, there is need to understand social, economic, cultural, and political factors when addressing complex problems (Bennett, Roth, Klain, Chan, Christie, et al., 2017; Bennett, Roth, Klain, Chan, Clark, et al., 2017; Zimmermann et al., 2020). This together with effective wildlife governance principles (e.g., Decker et al., 2016) will be required to prevent conflicts escalating and becoming increasingly difficult to resolve (Cusack et al., 2021; Zimmermann et al., 2020).

Research on HWC commonly leans toward identifying practical solutions to HWC (Pooley et al., 2017). This focus upon solving problems creates a negative framing of HWI and thus suggests a need for interventions that supply a “fix” (Pooley et al., 2017), but it fails to appropriately respond to the complexities of relationships between humans and wildlife (Bhatia et al., 2020).

Coexistence provides a more positive framing, with its focus on sustainable processes of wildlife and humans living together (Frank & Glikman, 2019; Pooley et al., 2021). *Coexistence* as a term is rarely defined (Knox et al., 2021) and is often used to mean tolerance of wildlife or human acceptance of negative impacts (Brenner & Metcalf, 2020). Glikman et al. (2021), however, suggest tolerance, acceptance, and coexistence exist within a hierarchy, with the latter taking precedent because it also enables benefits for people and wildlife to be derived.

One of the most accepted definitions of coexistence (developed from work exploring human–carnivore coexistence but applying across taxa [Carter & Linnell, 2016a]) is Carter and Linell's (2016b, p. 575):

…a dynamic but sustainable state in which humans and [wildlife] co‐adapt to living in shared landscapes where human interactions with [wildlife] are governed by effective institutions that ensure long‐term [wildlife] population persistence, social legitimacy, and tolerable levels of risk.

The authors argue coexistence is something more than co‐occurrence within a space; rather, it is a process that is active over time. By *coadaptation*, they refer to humans and wildlife “changing their behavior, learning from experience, and pursuing their own interests with respect to each other” (which in certain contexts may require human interventions) (Carter & Linnell, 2023, p. 1). Hence, interactions among ecological, cultural, and social factors are acknowledged (Hill, 2021), which has relevance to research and operationalization in practice (Carter & Linnell, 2016a). Effective institutions for governance are described as flexible and encompass “formal and informal rules that govern human behavior” (p. 575); participatory processes are given as an example of a decision‐making framework that supports inclusivity and social legitimacy.

Frank and Glikman further this thinking with their proposed conflict‐to‐coexistence continuum (Frank, 2016; Frank & Glikman, 2019) in which HWC and coexistence are linked. They do not consider them separate entities. The spectrum spans from the extreme negative, through neutral interactions, to the extreme positive. Conflict and coexistence are not static points on the spectrum but movable entities that can exist anywhere along it and fluctuate throughout it. It is even possible for different social groupings or contexts to exist at different points simultaneously. It is recognized that benefits and negative impacts may be unevenly distributed or understood differently among people (Carter & Linnell, 2023; Frank & Glikman, 2019). Conflict and management are integral facets of coexistence (Hill, 2021; Pooley et al., 2021), and the continuum reflects this while encouraging shifts toward the positive coexistence end, providing an aim for HWC management and representing HWI in a goal‐orientated sense.

We argue that Frank and Glikman's continuum together with Carter and Linnell's ideas on coexistence provides a powerful lens through which to examine HWI in reintroduction contexts. Reintroductions are human‐initiated, socioecological processes in which positive and negative interactions between people and wildlife or between people about wildlife may arise to differing levels at different times. The long‐term presence of the reintroduced species must be a central objective of reintroductions (Carter & Linnell, 2016b). We contend that reintroductions create unique contexts with specific features that have bearing on the ability to facilitate coexistence and that a recognized branch of coexistence for reintroduction is vital.

## DEFINITION AND NOMENCLATURE OF *RENEWED COEXISTENCE*

In reintroductions, the species being released has previously been present and will likely have lived alongside people. This may have been sustainably until extirpation through actions of nonlocal people, or in an unsustainable relationship with extirpation caused by actions of people locally. Over time, people living in the locality develop new knowledge and experience of the landscape, absent of the species, consequently losing knowledge of sharing the landscape with the species over time—a phenomenon known as the “extinction of experience” (Whitehead & Hare, 2025). In the intervening time before reintroduction, localities become increasingly anthropogenic (e.g., through urbanization or changing landscape practices) (Nyhus, 2016). Humans may then experience “shifting baseline syndrome” (Pauly, 1995), whereby there is an acceptance of ecosystems in their degraded state as the baseline natural state (Vera, 2010).

When reintroductions occur, they challenge accepted baselines and new HWIs occur with and between people and the reintroduced species in socioecological contexts (Auster et al., 2022a). Humans are integral to the coadaptation process if there is to be coexistence following release (Auster et al., 2022a; Marino, Crowley, et al., 2024). This can result in an expectation being placed upon those people to act or behave in ways that may be new to them (Auster, Barr, et al., 2020; Thulin & Röcklinsberg, 2020), particularly if the species has large effects, as large carnivores can do (Carter & Linnell, 2016b; Niemiec et al., 2021; Redpath et al., 2017). Accordingly, the reintroduced species must coadapt to live within modern‐day, anthropogenically modified landscapes as its population grows and disperses on the trajectory toward its socially accepted or ecological carrying capacity (and the regulation phase of population growth) (Gigliotti et al., 2000; Hull et al., 2023; Minnis & Peyton, 1995).

Auster et al. (2022a, p. 14) first used the term *renewed coexistence*:

…coexistence that is specifically associated with a reintroduced species, thereby one which was present in the landscape historically, but which will likely be a ‘new’ presence for the humans living in the locality post‐release.

The taxonomy of renewed coexistence was chosen to highlight fundamental underpinnings of reflecting on the past to look forward to future coexistence—inclusive of coadaptation.

As specified at the time, Auster et al. (2022a) intentionally included the term *coexistence* because they recognized the wealth of HWI research and experience that came before, from which much knowledge is applicable to the reintroduction context (and vice versa). We thus consider renewed coexistence a branch of coexistence that recognizes aspects that are unique to the context through the application of the term *renewed*.

*Renewed* was chosen because using the prefix *re*‐ acknowledges there may have been a historical relationship between humans and the reintroduced species and ‐*new* acknowledges the sense of newness behind the species presence in the landscape for the humans who live there now—particularly in cases where the species has been absent for some time and people are subject to extinction of experience.

Renewed coexistence frames reintroduction as a coadaptive process through which sustainable HWI must be fostered. Drawing on coexistence accounts for the social, political, cultural, and philosophical contexts within a setting (Glikman et al., 2021; Pooley, 2021) and emphasizes human understandings and relationships with the reintroduced species over time as it returns.

We considered the practical application of renewed coexistence in seeking to show that our conceptual reframing offers the following two key contributions relevant to research and practice.

First, underpinned by the conflict‐to‐coexistence continuum, renewed coexistence provides a goal‐orientated framing for reintroductions to enable sustainable outcomes (i.e., coexistence in the longer term, postproject). This framing influences reintroduction processes by encouraging strategic, futures‐oriented thinking toward sustainable coexistence from the outset.

Second, translocation science has principally focused on the feasibility, planning, and implementation phases. Renewed coexistence highlights that coadaptation continues thereafter through a newly recognized transition phase, by which we mean a period in which the species transitions from being considered new and reintroduced to a species that is familiar and considered wild. There is little research so far exploring such a transition or ways to support this process. With this conceptualization, we sought to encourage efforts to fill this research gap and inform improved reintroduction practices.

## APPLYING RENEWED COEXISTENCE

**FIGURE 1:**
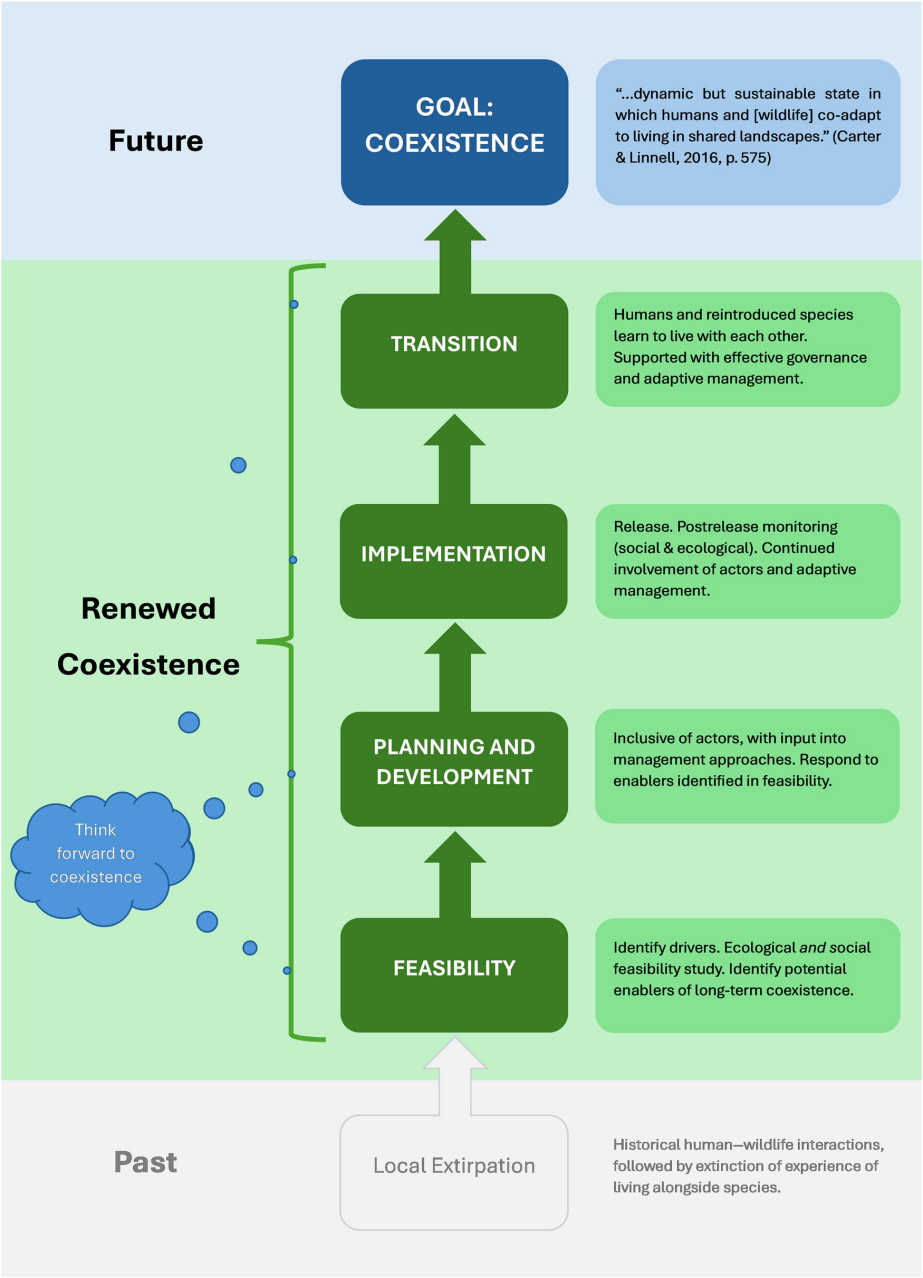
Renewed coexistence as could be applied in practice to animal reintroduction.

**FIGURE 2:**
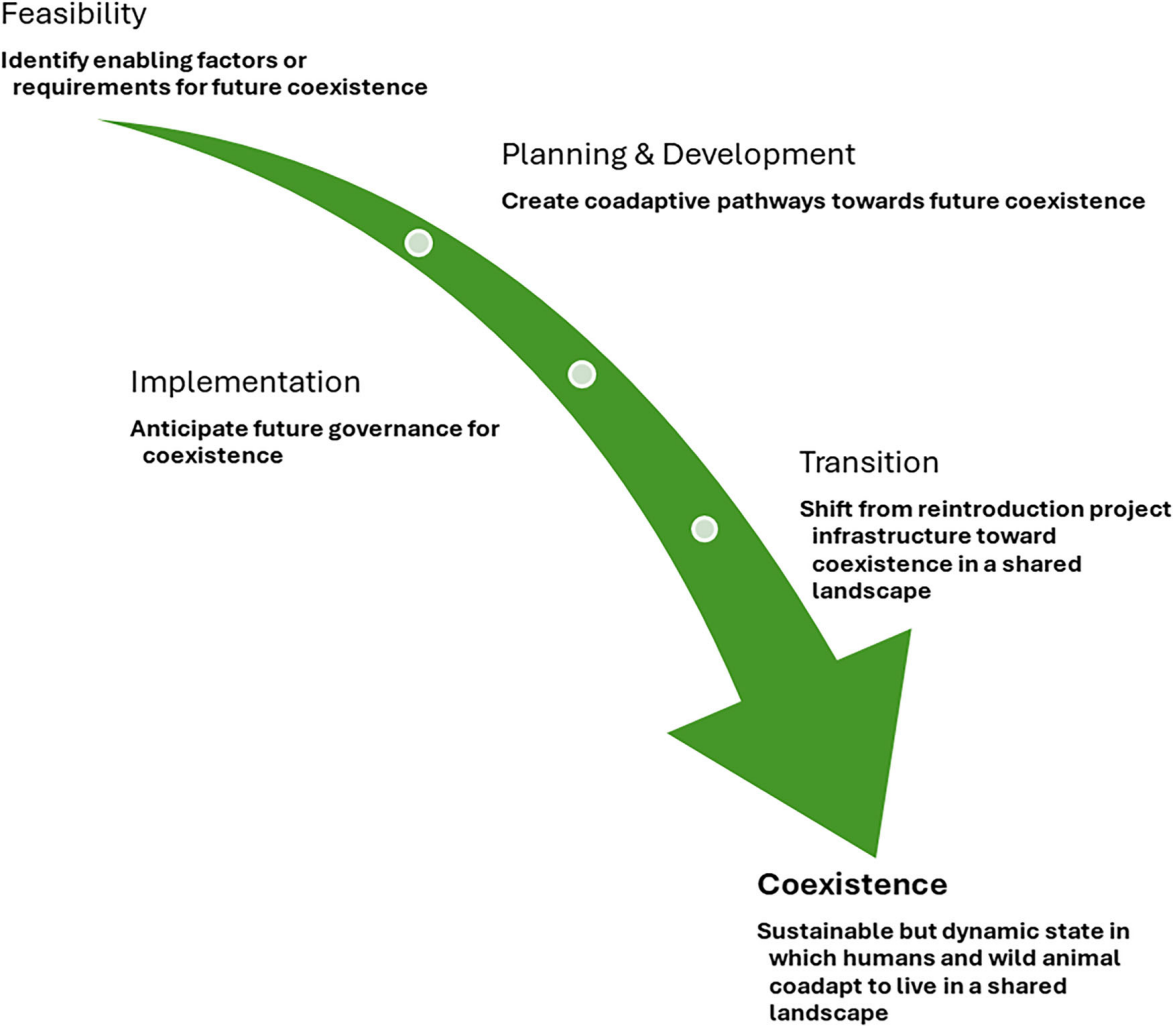
Integration of futures‐oriented thinking in the steps toward coexistence between people and (reintroduced) animals.

To demonstrate the contribution of renewed coexistence, we devised a theoretically ideal illustration of the process (Figure 1). We considered 4 broad phases: feasibility; planning and development; implementation; and transition. Although discussed separately, these phases occur through time and factors within them overlap. We contrived what we call practice notes to support practitioner reflections on how renewed coexistence can inform futures‐oriented reintroduction approaches, supported by Figure 2 and a subsequent real‐world example. However, because reintroductions are species and context specific and this is a conceptual discussion, we did not devise a comprehensive list of prescriptive recommendations. For further guidance, we recommend, for example, Auster et al. (2022a), Consorte‐McCrea et al. (2022), Glikman et al. (2023), IUCN SSC (2013), Marino et al. (2025), and Niemiec et al. (2021).

Renewed coexistence is a conceptualization of the transition to coexistence. This does not mean therefore that a strict step‐by‐step process through set phases must be followed to qualify. Renewed coexistence may be achieved in a multiplicity of ways, and principles may be applied at different phases. For example, in some contexts, prior reintroduction attempts may have been made and projects reorientate to integrate social dimensions. In these instances, the ability to renew coexistence may be challenged by prior events yet remain possible.

## Feasibility

Reintroductions commence against the backdrop of the present‐day, socioecological landscape. The idea is usually conceived by a primary actor (whether individual or organization), although it can arise from a codesign or collaborative process among a group of interest holders, the local community, or Indigenous peoples (Rayne et al., 2020; Treves et al., 2009). Reintroduction projects may reflect one or several drivers—such as ecological restoration, to inspire nature connection, or an ethical drive to restore native species (see Marino et al. [2025] for discussion on drivers).

Whatever the motivation, best practice is for the feasibility study to explore whether reintroduction is possible (IUCN SSC, 2013). Ecological feasibility is essential, yet so is social feasibility, and despite inclusion in the IUCN guidelines (IUCN SSC, 2013), this is often overlooked.

Dando et al. (2023) undertook a systematic review of 550 translocations and found social feasibility is often absent or tokenistic. They suggest barriers to uptake include resource limitation, lack of knowledge of how to undertake it, and prioritization of ecological factors. They argue social feasibility should take place in the early phases of reintroduction if findings are to influence subsequent phases meaningfully. Fewer social challenges arose in projects where social factors were appropriately addressed. We agree and further advocate for social feasibility to be prioritized at the outset with equal weight as ecological assessment (Auster et al., 2025) to reflect the socioecological system in which reintroductions take place.

The IUCN guidelines list some facets to include, such as assessing positive and negative impacts for local people and establishing engagement mechanisms (IUCN SSC, 2013). This is particularly important when those advocating coexistence with the reintroduced species may not themselves experience future interactions (König et al., 2020).

We suggest that, too often, social feasibility assessments—where they exist—are limited in scope and overfocus on impacts of reintroduction (and rarely compared with impact(s) of not doing so). Although important, assessments must go further. Social feasibility should interrogate findings for informative reflections upon which to build subsequent phases—reflecting the needs of both those who may be most affected and the wider communities (Dando et al., 2023). This may include identifying factors relevant to the development of governance mechanisms and interventions that respond to cultural understandings and factors that enable benefits to accrue (Niemiec et al., 2021).

### Feasibility practice note

Position social feasibility investigation alongside ecological investigation to meaningfully engage local people early, respect cultural contexts, and identify enabling factors or requirements for future coexistence.

### Example of using Q‐methodology in social feasibility

In 2014, Vincent Wildlife Trust began a pine marten (*Martes martes*) recovery program to restore viable populations in Wales (and England). Although public opinion surveys engage large numbers of people, they are limited in their ability to reveal nuanced perspectives or minority viewpoints (Bavin et al., 2020; Dando et al., 2023). Therefore, to develop rich insights within social feasibility for a Welsh reintroduction, Bavin et al. (2020) used Q methodology, which identifies and interprets shared perspectives within a context (methodological details in Watts & Stenner [2012]). The method enabled 4 nuanced perspectives to be identified: 3 supportive (with varying priorities, one more qualified) and one opposed (with concerns about impacts). From this rich understanding, areas of consensus could be identified (e.g., agreement that if pine martens had a negative impact on invasive gray squirrels [*Sciurus carolinensis*], there would be economic and ecological benefits), and understanding was gained of how wider issues influence perspectives (e.g., in the opposition viewpoint, preferences for traditional predator management practices interacted with concerns). Bavin et al. (2020) concluded that “by identifying diverse stakeholder perspectives and acknowledging the background and legitimacy of each, practitioners can reduce the potential for affected people to feel marginalized, promote inclusivity, and encourage a more democratic approach to conservation” (p. 1127).

## Planning and development

Often, the first social efforts occur when reintroduction is already being planned and proponents seek to rally support (Dando et al., 2023). Although gathering support is valuable, we consider doing so without concurrent and meaningful involvement of interest holders to be insufficient to meet potential future social challenges. It may already be late because psychological reactance to a proposal can arise where parties believe there has been inadequate opportunity for input and perceive that a project infringes upon their activities (Bavin et al., 2023; Lüchtrath & Schraml, 2015).

Planning should instead reflect findings of both social and ecological feasibility assessments, and plans should be developed through meaningful, holistic engagement that responds to challenges and enables opportunities (Auster et al., 2022a; Auster, Puttock, et al., 2020; Gaywood, 2024). Herein, this means a process in which interest holders are represented, involved early, have opportunity to influence project design, and communication is transparent (Consorte‐McCrea et al., 2022; Glikman et al., 2023). For example, much as Carter and Linnell (2016b) suggest, arguments are often made for participatory approaches whereby interest holders are actively involved in collaboration (Treves & Santiago‐Ávila, 2020; Treves et al., 2009). Where this is possible, local, traditional, or indigenous knowledges are more likely to be respected and integrated into planning design (Bennett, Roth, Klain, Chan, Christie, et al., 2017; Jolly & Stronza, 2025; Rayne et al., 2020) and trust is more likely to be built—perhaps even leading to a sense of ownership in a proposal (Auster et al., 2022a; Marino, Crowley, et al., 2024).

Significant challenges exist that mean such collaboration may sound idealistic rather than realistic. Some unsupportive of reintroduction on principle may be unwilling to engage in dialogue (Auster et al., 2022a), or there may be a lack of trust between groups from disagreements over prior events (e.g., earlier attempts to reintroduce a species, illegal releases of individuals, or other initiatives involving similar social groups) (Coz & Young, 2020; Lüchtrath & Schraml, 2015; Whitehead, 2025). Pragmatically, participatory planning may not be possible in all cases; yet, we advocate for genuine attempts to involve diverse voices.

We have observed that dialogue often centers on possible impacts of reintroduction, contributing to polarized disagreement and organized opposition surrounding binary proceed‐or‐not‐proceed decisions. There is far greater capacity for relationship building and opportunities to find consensus in discussion around the how of reintroductions, such as around management frameworks that could be put in place when reintroductions occur (Auster et al., 2022a) (perhaps alongside discussions on alternative approaches to achieving the same objectives where decision‐making suggests reintroduction should not progress [Auster, Frith, et al., 2023b; IUCN SSC, 2013; Marino et al., 2025]). Although disagreement may remain (many HWCs are between people about wildlife management [Marshall et al., 2007; Redpath et al., 2015]), we do not necessarily mean reaching unanimous agreement because not all conflicts can be resolved. Rather, we argue for efforts to collaboratively develop management approaches and establish governance systems that can support those negatively affected while enabling opportunities (Auster et al., 2022a; Brazier, Puttock, et al., 2020).

### Planning and development practice note

Design socially inclusive processes that reflect local socioecological contexts, enable community input, and create co‐adaptive pathways toward future coexistence.

### Example of meaningful engagement in development

Since 2018, Natural England has been working to reintroduce hen harriers (*Circus cyaneus*) in southern England. Because entrenched HWC exists among actors about hen harriers in the United Kingdom, the team sought to involve and have dialogue with these actors to reduce potential for future escalation from reintroduction. They ran a localized engagement program with interest holders to address concerns and discuss management issues. From participant observation and interviews, Marino, Crowley, et al. (2024) detail how pretranslocation engagement activities initiated collaborative processes, facilitated social learning, and could lead to positive outcomes, such as trust between interest holders. Marino, Crowley, et al. (2024, p. 7) argue that there is a need for practitioners to reflect on how projects may interact with existing circumstances and conflicts, which “is especially crucial during the pre‐translocation stage, where in the absence of concrete implemented measures and outcomes, stakeholder perspectives are more likely to be shaped by existing knowledge of current conservation issues at various scales” and that “a key pre‐translocation process such as stakeholder engagement has transformative potential when qualitatively well designed and implemented” (p. 7).

## Implementation

Animal releases then take place ideally accounting for ecological, animal welfare, and genetic considerations (IUCN SSC, 2013). Throughout implementation—with the establishment and initial growth of a population and associated postrelease monitoring—the continued involvement of people remains vital. Sustained, meaningful dialogue with key parties will help maintain trust; awareness raising can garner public support (Sampson et al., 2020); and employment of adaptive management strategies can demonstrate how conflicts can be addressed (Auster et al., 2022a).

### Implementation practice note

Nurture trust with socially inclusive, coadaptive management that anticipates future governance for coexistence.

### Example of continued involvement during implementation

In the Black Forest, Germany, a lynx (*Lynx lynx*) reintroduction is underway. In the 1980s, a release proposal by a conservation group faced opposition from farming and hunting groups, leading to a court judgment that prevented release at that time (Herdtfelder et al., 2021). Lüchtrath and Schraml (2015) used focus groups to understand hunter perspectives and found that concerns were about not just the lynx but also about defense of their social identity as a group. Since then, the Working Group Lynx has fostered collaboration (Herdtfelder et al., 2021), and as a testament to long‐term efforts, the State Hunting Association (Landesjagdverband Baden‐Württemberg) became one of several partners in a project that released the first lynx in 2022. The working group, now Lynx & Wolf Working Group, continues to meet. A member of the Hunting Association said, “The cooperation between the hunting community and the project has been excellent so far” (Ministerium für Ernährung, Ländlichen Raum, & Verbraucherschutz Baden‐Württemberg 2024).

## Transition

Although iterative monitoring and management efforts may continue, project evaluations usually take place after a period to assess multiple outcomes (as recommended in IUCN Guidelines Annex 8). In many cases, the project time frame ends and outcomes are deemed to have succeeded or failed in relation to ecological factors, such as the establishment of a self‐sustaining population (Marino, McDonald, et al., 2024; Niemiec et al., 2021; Seddon et al., 2007).

Valuable efforts to develop best practice guidance for inclusion of human dimensions in reintroductions have also been tailored to the traditional project timescale, concluding with evaluation. For example, Niemiec et al. (2021) discuss a framework to incorporate social science in planning, with the final phase being evaluation. Although HWIs will have commenced from the outset and developed in earlier phases, animals and humans remain in the landscape after the reintroduction and far beyond the funding cycle; HWIs continue and new interactions arise as humans and the introduced species continue to live with one another and as the species’ population grows.

This period of transition, from reintroduction to coexistence, has received minimal attention. Renewed coexistence draws attention to the urgent need to further understand dynamics within this time frame and how this understanding can inform the entire reintroduction process.

In interviews with individuals who reported conflicts with beavers (*Castor fiber*) in the River Otter Beaver Trial in England (Brazier, Elliott, et al., 2020), a 5‐year project with a defined end point, we identified a need for certainty into the future. Uncertainty about the ability to access support or to manage negative outcomes after the project time frame generated worry, which in turn affected decision‐making (Auster, Barr, et al., 2020). In an approach seeking to renew coexistence, strategies that support people learning to live with reintroduced species should be considered prior to implementation as far as possible.

There is need for realism here. It is impossible to account for every possible interaction and unreasonable to expect practitioners will have full autonomy—for example, legislative policies or political directives are not necessarily in the hands of practitioners or communities (Auster et al., 2022a). Yet, alongside communication of these limitations to help manage expectations, plans that are proactively developed (as far as possible) to capture and respond to newly occurring HWIs can support best efforts to enable coadaptation as the reintroduced animal's population grows and transitions from the subject of a project to part of the local fauna (Auster et al., 2022a).

In these same interviews (Auster, Barr, et al., 2020), we identified a sense among some people of a perceived link between the presence of the species and, in the words of one participant, the “people who put them there.” Among individuals with this perspective, this translated into a sense of accountability for conflicts experienced and a higher expectation of a management response than expected for other wildlife. These findings emphasize key questions that remain underresearched—for example: when and for how long is it appropriate to hold practitioners accountable for the actions (or welfare) of autonomous animals; how can people be best supported as they learn to live with the species; how long will it take for the species presence to become socially normalized and how is normalization best achieved; how can people be weaned off project infrastructure (Consorte‐McCrea et al., 2022); and what governance frameworks best support transition.

We believe this to be vital knowledge to develop, aiding approaches to support coexistence from the reintroduction context and into the sustainable and coadaptive state of living together.

### Transition practice note

Maintain relationships and support continued coadaptation, while shifting from reintroduction project infrastructure toward coexistence in a shared landscape.

### Example of a mechanism for transition

Eurasian beavers (*Castor fiber*) are being reintroduced to England. Following a 5‐year project, Devon Wildlife Trust and partners have coordinated Beaver Management Groups as fora to engage local people and familiarize them with beavers and management interventions (Auster et al., 2022b). Auster, Puttock, et al. (2023) used interviews to capture lessons from these groups and found the species‐specific management groups were not fixed structures but rather adaptive processes that supported people as they learned to live with the species. Membership in these groups changed as new people attended meetings where new issues arose, and others ceased attending when they learned to coexist. As resource‐intensive structures, the authors concluded that [s]pecies‐specific management groups could be beneficial in reintroductions by providing a moving “front‐line” for involving local actors and “familiarizing them with reintroduced species” but that “as people learn to live with reintroduced species, the need for species‐specific management groups may reduce and their role could be scaled back over time” with day‐to‐day management integrated into existing structures (Auster, Puttock, et al., 2023, p. 5).

## COEXISTENCE AS THE GOAL

Although we introduce coexistence here as though it is a phase in the renewed coexistence process, it is not. The sustainable, dynamic, coadaptive state of living together (Carter & Linnell, 2016b), inclusive of a self‐sustaining population of the reintroduced species (Marino, McDonald, et al. 2024), is the end goal that succeeds renewed coexistence and that renewed coexistence approaches work toward.

Conceptually, we define the passage into coexistence as the point at which the reintroduced species is considered a wild animal that is a natural part of the environment, rather than as a reintroduced species with a direct link to the “people that put them there.” Thus, coexistence between humans and the species has been renewed, and they continue forward into a coadaptive state of living together. Identifying exactly when this point has been reached may be difficult because it is unlikely to materialize as a visible moment, and different people may consider the animal to be a natural part of the environment at different times (Auster et al., 2022b). Yet, theoretically, when this point is reached, the unique aspects of the reintroduction context no longer apply, and humans will coexist with the species as native fauna.

## CONCLUDING REMARKS

Where reintroductions take place, coexistence should be the aim—above and beyond simply the return of an animal into a landscape. Human–wildlife coexistence can enable a successful reintroduction as a sustainable endeavor. Considering reintroductions as processes of renewed coexistence provides a necessary goal‐orientated framing to encourage research and enable practitioners to ensure HWIs are built into socioecological projects from the outset, throughout, and beyond the formal end of the project. Finally, we provided fresh insight into the period of transition as a vital but understudied phase. This phase requires attention so that humans and reintroduced animals can be better supported as they coadapt to each other, until they coexist.

## AUTHOR CONTRIBUTIONS

R.E.A. conceived the idea, conceptualized renewed coexistence, and led the writing of the manuscript. S.B. and R.B. supervised an earlier PhD program undertaken by R.E.A., which inspired initial thinking for this piece. All authors discussed the conceptualization and contributed critically to drafts.
